# Prophylaxis and hemophilia care in LATAM: Baring it all—Highlights from the CLAHT 2021 symposium

**DOI:** 10.1002/jha2.503

**Published:** 2022-08-10

**Authors:** Giovanni G. DiMinno, Lida Milena Araujo Cabrera, Nancy Loayza Urcia, Raul Bordone, Carlos Martinez Murillo, Juan Carlos Beltran, Prasad Mathew

**Affiliations:** ^1^ Centro Hub per le Malattie Emorragiche Congenite e le Trombofilie Università degli Studi di Napoli “Federico II” Napoli Italy; ^2^ Fundación Clínica Infantil Club Noel and Oncólogos Asociados de Imbanaco Cali Colombia; ^3^ Hospital Dos de Mayo Lima Perù; ^4^ Centro de Tratamiento Hemofilia Córdoba Córdoba Argentina; ^5^ Hospital General de Mexico Mexico City Mexico; ^6^ Kedrion de Colombia SAS Centro Empresarial Arrecife Bogota Colombia; ^7^ Kedrion USA Fort Lee New Jersey USA; ^8^ Presbyterian Hospital Albuquerque New Mexico USA

**Keywords:** access, CLAHT, hemophilia, Latin American, pd‐FVIII, prophylaxis

## Abstract

A large group of countries constitute Latin American (LATAM) countries, where hemophilia care is as varied as the landscape of this region. To better understand the care provided to persons with bleeding disorders, especially hemophilia, a symposium was organized as part of the CLAHT Congress 2021 in Colombia to highlight the issues of hemophilia care and challenges faced by persons with hemophilia in four LATAM countries, Colombia, Peru, Argentina, and Mexico. A summary of the symposium is provided. Four clinicians highlighted the issues in their own country, the status, and the path forward to bring the standard of care to the international level in each of these countries. The geography of the country, the health infrastructure, and the resources available are obstacles in these countries to provide state‐of‐the‐art care to the bleeding disorder community. However, depending on the country, its infrastructure and the resources available, progress is being made to upend the care provided. Indeed, the care of persons with hemophilia has been greatly improved, including personalized prophylaxis. The information summarized here first emphasizes how the geography of a country and the different healthcare infrastructures play a major role in how care is offered. It also provides a path for other countries to evaluate these issues in their own realities. In parallel, these data provide hope to many developing countries; despite obstacles, strides can be made in the care of the bleeding disorder community.

## 1 INTRODUCTION

In 2018, in a paper summarizing the results of a survey taken among Latin American (LATAM) countries, Boadas et al. [1] identified issues in advancing the principles of care for persons with hemophilia (PWH) and suggested alternatives that should be implemented to improve the quality of life of PWH in the region. Progress is being made with the introduction of national registries for PWH in eight countries, the creation of integrated care centers, at least in larger cities, and the availability of access to home treatment under special conditions. However, access to factor concentrates, and restrictions on the use of prophylaxis and provision of care in smaller cities remain a challenge. A recent article from HemoHermanos [2] stated, “Deaths, irreversible health damages, delayed treatments, and obstacles to accessing medicine are part of the landscape”—giving a view from the patient's perspective.

To explore this sentiment, a symposium was organized at the CLAHT (*Grupo Cooperativo Latino Americano de Hemostasis y Trombosis*) [3] 2021 virtual Congress in Colombia with the purpose of looking at the current state for prophylaxis and hemophilia care in these countries. Given the time available for the symposium, four countries provided an overview from the treater's perspective. This paper summarizes highlights from this symposium, which showcased the current state of care in these four countries. The symposium was chaired by Prof. Giovanni DiMinno from Italy, while the speakers included hemophilia treaters from Colombia, Peru, Argentina, and Mexico.

## 2 SYMPOSIUM SUMMARY

The stage was set by the chair, highlighting the availability and improvements in factor replacement therapy at a global level, as well as the benefits of prophylaxis in preventing and maintaining joint health. The value of pharmacokinetics (PK), guided dosing, and value of personalized prophylaxis were also mentioned. Highlights from the third edition of the World Federation of Hemophilia (WFH) guidelines [4] for the management of hemophilia provided the background for framing the questions to the speakers regarding the status of hemophilia care and prophylaxis in their country, challenges, and future solutions in meeting these challenges while elucidating the role of desmopressin (DDAVP) as part of the management of hemophilia and Von Willebrand disease (VWD).

## 3 CHALLENGES FOR HEMOPHILIA PROPHYLAXIS AND DDVAP USE IN COLOMBIA (DR. LIDA MILENA ARAUJO CABRERA)

### 3.1 Health system

**FIGURE 1:**
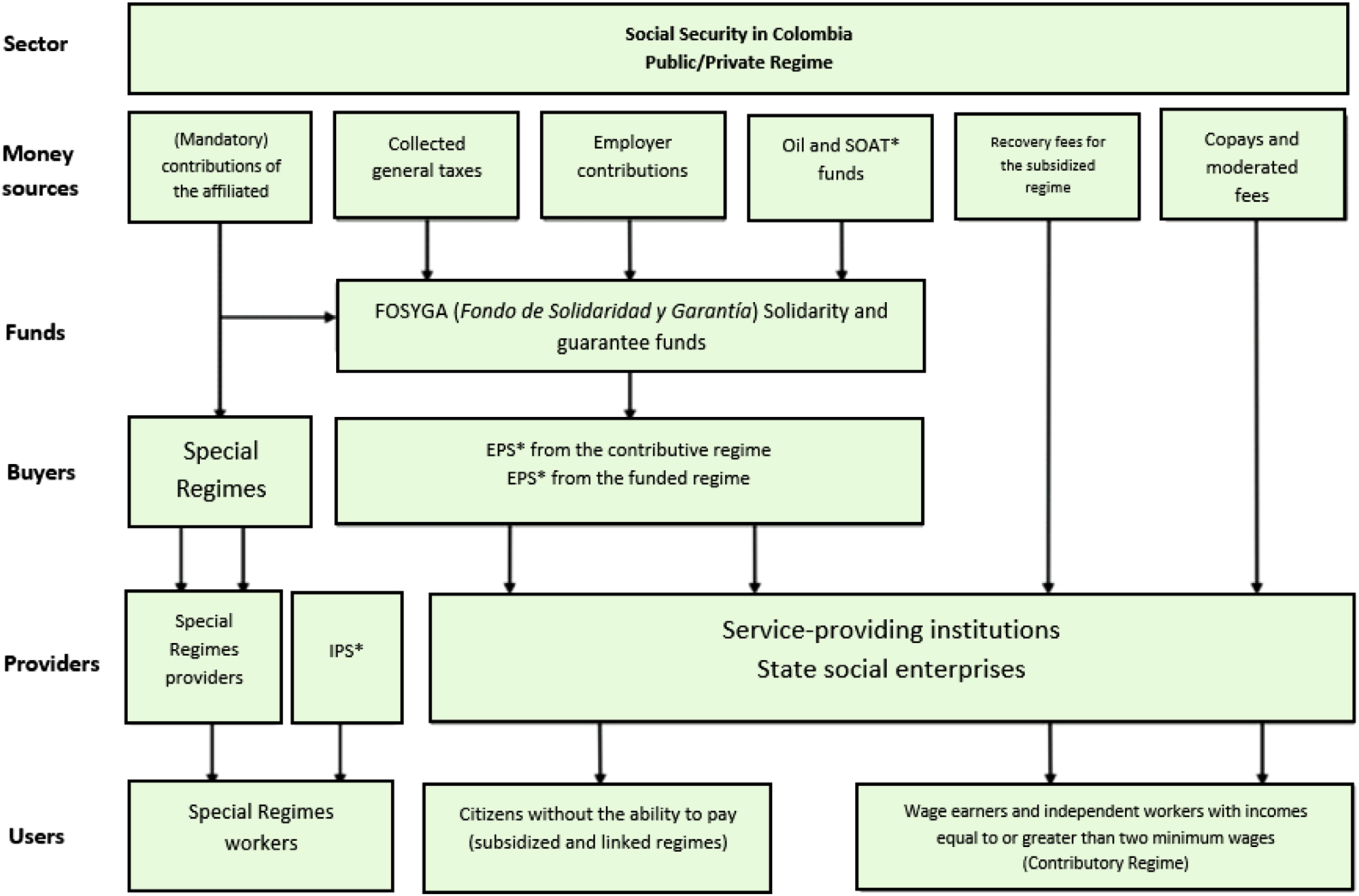
The Colombian health system. EPS, Entidades Promotoras de Salud or Health Promoting Entities; SOAT, Seguro Obligatorio de Accidentes de Tránsito, or Mandatory Traffic Accident Insurance; IPS, Instituciones Prestadores de Salud or Health Provider Institutions. https://www.scielosp.org/article/spm/2011.v53suppl2/s144‐s155/

The Colombian health system, created in 1993, consists of both the public and the private sectors that send contributions from both taxpayers and the government to a solidarity and guaranty fund called FOSYGA (Figure 1), which controls the resources for the healthcare system. Within the private sector, the funds are distributed and managed by administrative entities of health benefit plans (EAPB), where the employer covers 30% and the employee 70% of the cost of healthcare services. Within the public fund, there are also EAPBs; however, these are funded by the Colombian government and provide services to people with low economic resources. There is also a group of people who do not have an EAPB, such as street dwellers. Finally, there are special regimes that include the army and the teachers, who have the advantage of having no access restrictions to special drugs that require approval from a professional board.

### 3.2 Providers

These include special regime providers, service‐providing institutions, state social enterprises, and the IPS (*Instituciones Prestadores de Salud*) or “health provider institutions,” which include private clinics, public hospitals, and healthcare centers that provide medical care to all the patients (Figure 1).

### 3.3 Care for bleeding disorders

Diseases such as hemophilia and VWD are considered orphan diseases and receive special protection from the Colombian government; their healthcare is never denied. However, one of the issues that emerged was the finding that some health‐providing institutions tried to obtain financial benefits from patients being falsely reported as hemophiliacs or affected by VWD due to the high reimbursement cost of clotting factors. As a result, the Colombian government ordered a price regulation on these drugs and created a high‐cost account to carry out exhaustive surveillance of the number of existing patients with bleeding disorders in the country, who must have a diagnostic confirmation by laboratory tests and be fully verified by said entity.

### 3.4 Patients

**FIGURE 2:**
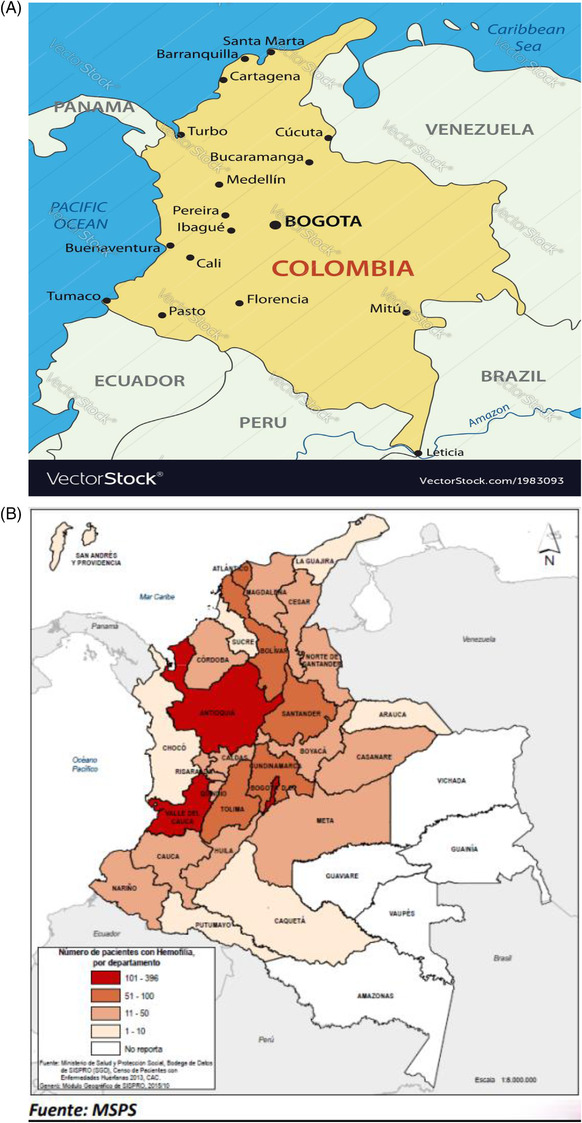
(A) The country of Colombia, with Bogoto the main capital city for hemophilia care. (B) Colombia: centers of hemaophilia in the different regions where patients are being seen

Ninety‐three percent of patients with hemophilia are cared for by a hematologist. There were 2863 patients with hemophilia A (82%) and B (18%) reported in 2020 (Figure 2, Table 1) [5], with the highest prevalence of hemophilia in the city of Bogotá (8.3/100,000 inhabitants). For hemophilia A, there was a male preponderance with the severe form (60%). In hemophilia B, most males presented with moderate severity (38%). Colombia is one of the few LATAM countries that guarantees access to prophylaxis even into adulthood. Indeed, 90% of patients with severe hemophilia A and B received prophylaxis in Colombia between February 2019 and January 2020 (Table 2).

**TABLE 1:** Colombia: demographic and clinical characterization (2020)

Variables	Hemophilia A	Hemophilia B	
Age	22.66 (12.43–34.40)	21.03 (12.73–32.52)	
Agency	*n* (%)	*n* (%)	Total (A and B), *n* (%)
Contributory	665 (55.79)	121 (53.30)	786 (55.39)
Funded	491 (41.19)	99 (43.61)	590 (41.58)
Exception	34 (2.85)	7 (3.09)	41 (2.89)
Special	0 (0.00)	0 (0.00)	0 (0.00)
Not affiliated	2 (0.17)	0 (0.00)	2 (0.14)
	100%	100%	100%
Severity			
Mild	12 (1.01)	3 (1.32)	15 (1.06)
Moderate	142 (11.91)	73 (32.16)	215 (15.15)
Severe	1.038 (87.08)	151 (66.52)	1.189 (83.79)
	100%	100%	100%
Presence of inhibitors			
Low response	81 (6.80)	10 (4.41)	91 (6.41)
High response	74 (6.21)	6 (2.64)	80 (5.64)
Negative	813 (68.20)	171 (75.33)	984 (69.34)
No test result	215 (18.04)	38 (16.74)	253 (17.83)
Test not performed	4 (0.34)	0 (0.00)	4 (0.29)
No data	5 (0.42)	2 (0.88)	7 (0.49)
	100%	100%	100%

*Note*: https://cuentadealtocosto.org/site/hemofilia/dia‐mundial‐de‐la‐hemofilia‐2021/.

**TABLE 2:** Colombia: characterization of treatment options (2020)

	Variables		
	Hemophilia A, *n* (%)	Hemophilia B, *n* (%)	Total (A and B), *n* (%)
Factor administered			
Factor VIII	807 (99.26)	0 (0.00)	807 (82.01)
Factor IX	0 (0.00)	169 (98.83)	169 (17.17)
rFVIIa	3 (0.37)	2 (1.17)	5 (0.51)
Emicizumab	3 (0.37)	0 (0.00)	3 (0.30)
	100%	100%	100%
Administration method			
Institutional	102 (12.55)	25 (14.62)	127 (12.91)
Domiciliary	494 (60.76)	86 (50.29)	580 (58.94)
Mixed	94 (11.56)	25 (14.62)	119 (12.09)
Self‐administered	92 (11.32)	28 (16.37)	120 (12.20)
No data	31 (3.81)	7 (4.09)	38 (3.86)
	100%	100%	100%
Venous access			
Peripheral	776 (95.45)	164 (95.91)	940 (95.53)
Central	6 (0.74)	0 (0.00)	6 (0.61)
Subcutaneous	3 (0.37)	0 (0.00)	3 (0.30)
No data	28 (3.44)	7 (4.09)	35 (3.56)
	100%	100%	100%
Dose (mean IU/kg/dose);range	26.50 (22.00–31.40)	29.40 (24.20–34.90)	
Prophylaxis frequency			
Once/week	28 (3.44)	13 (7.60)	41 (4.17)
Twice/week	146 (17.96)	135 (78.95)	281 (28.56)
Three times/week	606 (74.54)	19 (11.11)	625 (63.52)
Four times/week	22 (2.71)	1 (0.58)	23 (2.34)
≥5 times per week	4 (0.49)	0 (0.00)	4 (0.41)
More than 1 week interval	7 (0.86)	3 (1.75)	10 (1.02)
	100%	100%	100%

*Note*: https://cuentadealtocosto.org/site/hemofilia/dia‐mundial‐de‐la‐hemofilia‐2021/.

### 3.5 Effect of prophylaxis

Prophylaxis has been associated with a 60% decrease in the risk of inhibitor development compared to on‐demand treatment. Personalized prophylaxis in hemophilia depends on several factors, including severity, number of bleeds, presence of inhibitors or arthropathies, adherence to the therapy and treatment costs, as well as self‐management capacity. Table 2 shows the demographics and characterization of the treatments provided in 2020, with 56% of PWH receiving some type of prophylaxis. Of these, only 6% developed high‐response inhibitors. Hospitalizations for bleeding decreased from 19% in 2015 to 10% in 2020, and a 9% growth in medical care by the interdisciplinary team was observed, from 39% in 2019 to 48% in 2020.

### 3.6 Prophylaxis recommendations in Colombia

Initiating prophylaxis in hemophilia A and B with plasma‐derived products, at least for the first 50 exposure days, to reduce the risk of inhibitor development (from the SIPPET study data) [6]. The choice of the product used should be agreed upon together with the patient and their family after discussion about the benefits and limitations of each product. Continued ongoing education of the patient and family is of key importance. Finally, it is important to understand the difficulties of PWH that limit the use of prophylaxis and to encourage self‐infusion.

### 3.7 Limitations in Colombia

There are only a few institutions that are called referral centers where patients can receive integrated care, both outpatient and hospital care. Presently, small health provider institutions provide only external consultation services and then refer for hospital care to a health institution with a higher level of complexity. Many patients do not have such access, which represents an obstacle in their access to emergency care. Not all hemophilia programs have PK capabilities that can help with doses and frequency of administration in patients who would like to do self‐infusion at home. The prophylaxis cost of extended half‐life clotting factors, mimicking molecules (emicizumab) and bypassing agents is very high, with very low accessibility to these drugs compared to conventional clotting factors. Healthcare centers and institutions may not have all products at hand to provide for emergency care of patients who are on a particular product for prophylaxis, which often leads to administration of products different from what the patients are using. Laboratory results for factor VIII and IX levels in a patient with suspected hemophilia may take 1–2 weeks in low‐complexity clinics or hospitals due to a lack of resources. Thus, samples must be sent out to be processed by an external laboratory.

### 3.8 DDAVP and VWD challenges

In Colombia, desmopressin is available for intravenous administration only, and it is easily accessible. It is underutilized due to the difficulty of measuring the antigen levels and the activity of Von Willebrand factor (VWF) and FVIII in laboratories for the evaluation of this drug's effectiveness. Nasal DDAVP is not available in Colombia.

### 3.9 Limitations for the use of DDAVP in VWD

There are challenges for the diagnosis and classification of VWD due to limitations with the laboratories that process the samples. The results may take between 1 and 2 weeks, sometimes due to batching of samples for processing, to save on use of the reagents and reduce the costs of the tests. Healthcare entities do not authorize the performance of VWF multimers and platelet aggregation curves because they do not have contracts with health institutions that have specialized hematology laboratories to perform such tests. Even though DDAVP is approved and available in Colombia, its use is limited by lack of knowledge, insufficient experience in its application and limitations in the processing of laboratory samples.

### 3.10 Conclusions

Colombia has availability and access to factor replacement therapy, and prophylaxis from childhood into adulthood is provided. Extended half‐life products and nonfactor therapies are still not easily available. Plasma‐derived factors have scientific evidence of a lower development of inhibitors compared to recombinant products. It is recommended to start plasma‐derived factor application in recently diagnosed hemophilia patients at least for the first 50 applications to reduce the risk of developing inhibitors. Prophylaxis reduces acute bleeding events and chronic complications of hemophilia compared to on‐demand treatment. Availability and access to laboratory facilities for testing and monitoring remains a challenge in many cities and institutions. It should be considered to employ DDAVP in VWD with adequate care in its infusion and recommendations to avoid possible adverse effects.

## 4 SITUATION IN PERU (DR. NANCY LOAYZA URCIA)

### 4.1 Land

**FIGURE 3:**
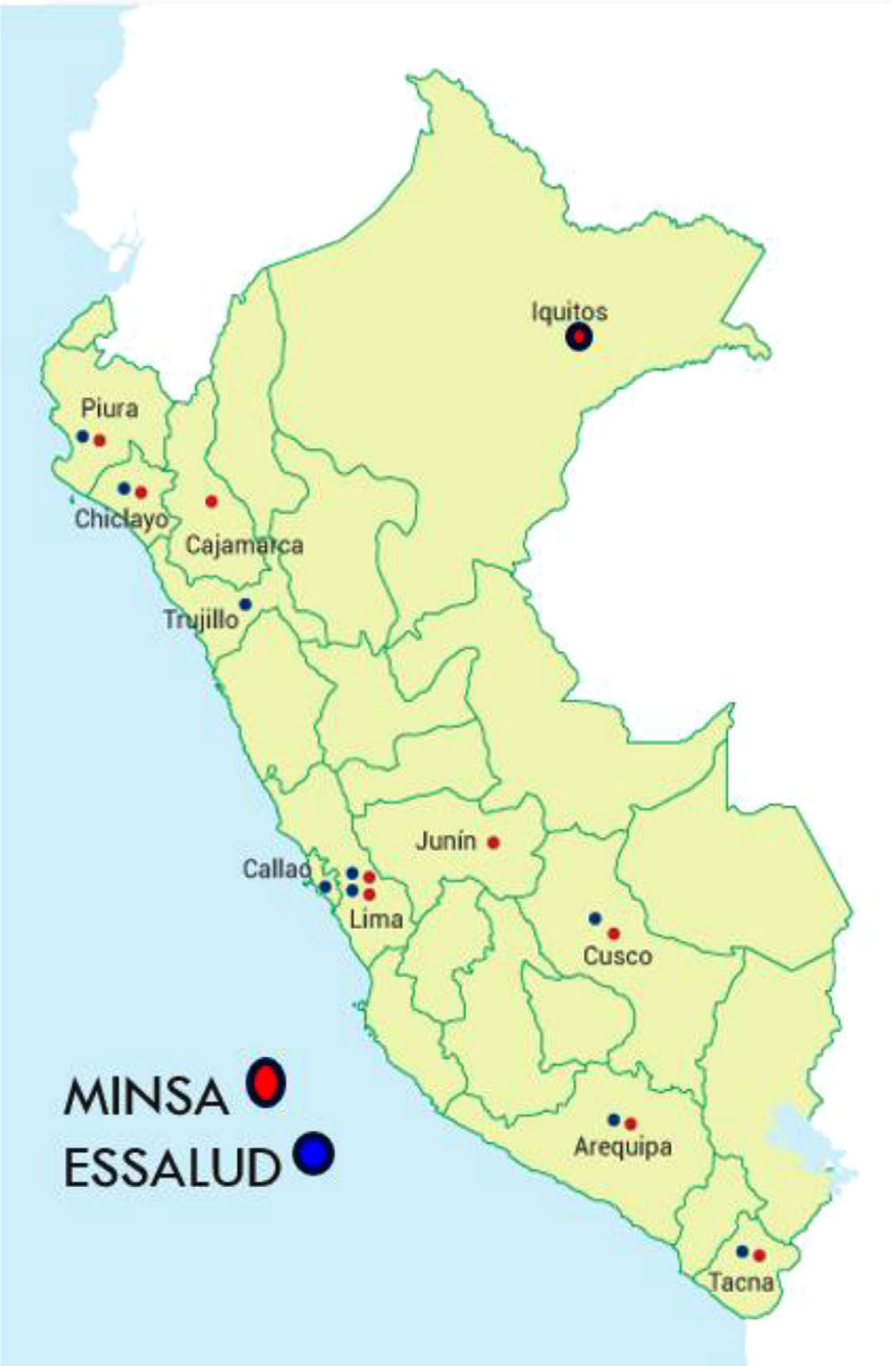
Peru: the land, treatment centers, and health care system

The country of Peru has an area of 1,285,220 km^2^ with a large territory and a population of 32,971,854 inhabitants, according to the last census. The concentration of the population on the coast is 52.6%; 38% are concentrated in the highlands and 9.4% in the jungle (Figure 3). The population is centered mainly in Lima, the capital city, with almost 30% of the overall population; in rural areas and especially in the jungle, the density of the population is very low. The country is divided into 24 regions, and the percentage of health expenses, the GDP (Gross domestic Product), is 3.3%. This is the last data taken in 2018, but it has not changed much in the last few years: it is one of the lowest in the region.

### 4.2 Health system

The health system is a mixed public–private system that generates some difficulties in the care of different pathologies, especially in the case of hemophilia. The Health Ministry Department (MINSA) provides the largest healthcare expenditure coverage, for approximately 50% of the population; the EsSalud workers contribute approximately 30% monthly; armed forces, police, and private forces account for another 10%, and the remaining 10% of the population does not have access to the health system and probably resides in the most remote areas of Peru.

### 4.3 Hemophilia care

Care for hemophilia patients started on June 4, 2011, when law number 29698 was approved. It defined the care and treatments available to people suffering from rare or orphan diseases of national interest and priority, including hemophilia and VWD. Before 2011, both the Health Ministry System and EsSalud treated hemophilic patients without providing a well‐defined structure for those pathologies. On February 22, 2019, the regulation on rare or orphan diseases was established, and on November 21, 2019, it was published, by ministerial resolution number 191075, the technical document listing all the rare and orphan diseases, where hemophilia and VWD were included. Thus, care for bleeding disorders in a structured manner in Peru is of recent origin. By establishing such a structure, the state could start investing and granting a budget for such pathologies, differently from what used to happen before.

### 4.4 Hemophilia treatment centers

There are 10 centers directly under the Ministry of Health. The first one was established in Lima, but in recent years, in the process of decentralizing (red dots), there are now 10 hemophilia treatment centers (HTCs) in Peru. EsSalud oversees nine centers, and the blue round one belongs to the armed forces centralized in Lima (Figure 3). There are a large number of regions where one cannot find any treatment center. Only 11 regions have treatment centers, and thus some sort of centralized hemophilia care is needed. Six regions have hospitals from both the Ministry of Health and EsSalud; however, there is no service exchange between them, which creates a serious problem for patient care due to lack of exchange of information for the different sectors involved. Thirteen regions do not have any HTC. This creates a huge burden since hematologists are not able to detect patients with hemophilia in such a large population and, even less, patients with VWD.

### 4.5 Laboratory diagnosis

This is also a serious problem since only a few treatment centers perform hemostasis tests routinely, specifically the coagulation profiles. Moreover, inhibitor tests for factors VIII and IX and studies for VWD are mainly centralized in Lima. Arequipa, a region located in the south of Peru, only measures factor VIII and IX levels. The remaining treatment centers, although able to provide care to hemophilic patients, do not have reference laboratories for diagnosis. This creates a serious problem, since suspected patients (based on their family history, or simply because doctors consider the diagnosis based on clinical manifestations) must travel to Lima for a diagnosis. Laboratories still use the clotting time method. Other coagulation methods are being tried, but they are still not in routine use in Peru. Another problem is that clotting factor tests are only carried out during the day shifts, therefore limiting the evaluation of factor assays during a 24‐hr period. This hampers the possibility of delivering a personalized approach to prophylaxis. This is mainly due to bureaucracy problems: each center makes its own reagent purchases independently. There is no centralization of purchases, and VWD studies are not carried out in the hospitals of the Ministry of Health but only in EsSalud hospitals in Lima, which provide diagnostic support.

### 4.6 Treatment issues

Peru has only plasma‐derived clotting factors. Recombinant factors are not yet available. Peru does not have extended half‐life clotting factors either, except the ones that come from donations from the World Hemophilia Federation. Prophylaxis in hemophilia A is performed in 80% of identified patients, and it is mandatory to start it as soon as the diagnosis is made, even in the first years of a child's life. For hemophilia B, only 50% of patients receive prophylaxis. This is lower than the percentage for hemophilia A due to the inconvenience of purchasing clotting factor IX by the hospitals of the Ministry of Health. Prophylaxis is not personalized due to logistical problems, mainly due to a lack of organization in establishing the availability of clotting factor tests 24 hrs in the different centers. EsSalud and MINSA manually fulfil public solicitations for the purchases of factor concentrates for VIII and IX. They do it separately, which makes it difficult to evaluate clotting factor usage. In 2020, purchases of clotting factor VIII in Peru were approximately 100 million and 12 million units for factor IX. Bypassing agents are those provided by EsSalud. The Ministry of Health has not acquired any bypassing therapy, which is a serious problem. Because it is not in the national petitioner, by not being in such a request document, the comprehensive health insurance of the Ministry of Health hampers purchasing of such products. Emicizumab has already been included in the registry for 2 years, but no purchase has been made yet. EsSalud has just authorized a special purchase of emicizumab for a patient with inhibitor.

### 4.7 Von Willebrand disease

There is an issue concerning its diagnosis and treatment by the Ministry of Health, since it is not under the national petitioner, while EsSalud has this capacity. Desmopressin is only used as a nasal spray in Peru, and the intravenous form is not available.

### 4.8 Hemophilia population

**FIGURE 4:**
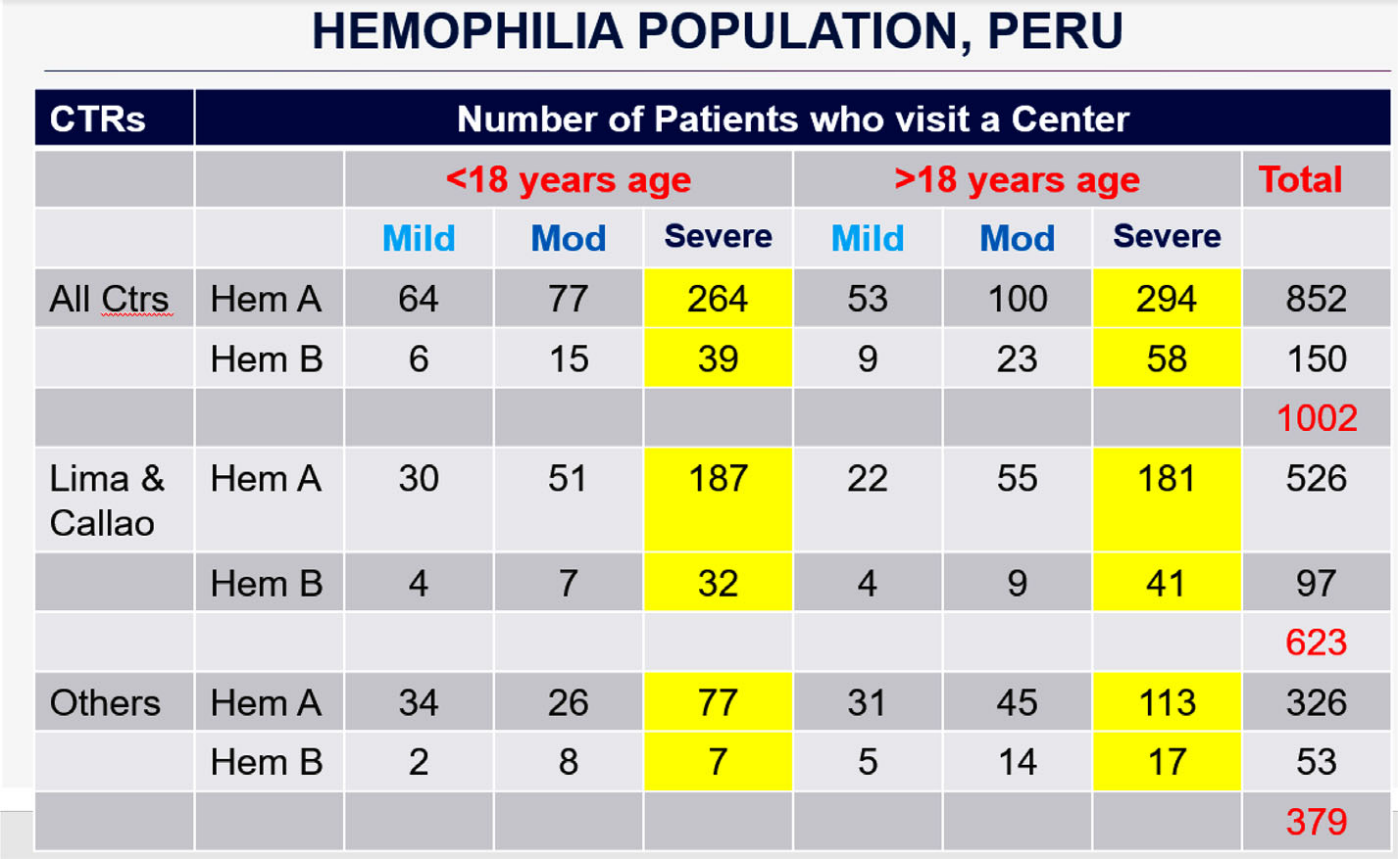
Distribution of hemophilia population in Peru. Project Hemophilia Peru 2018. Funded by Novonordisk—Sociedad Peruana de Hematologia

Based on the *Hemophilia Peru* project, a project in development since 2018, which unified centers under both MINSA and EsSalud, in the first census that included the centralized information, the total number of identified PWH is exactly 1002 patients (Figure 4). Data with regard to the types of hemophilia, the age of the patients and their origin are also being captured. As a result, it is estimated that there are 3200 people with hemophilia, and according to the census, only 31% of the patients have been identified. The great gap of patients who are not diagnosed yet, the remaining 69%, are probably spread in different parts of Peru. Therefore, there exists a tremendous challenge in continuing the identification process through the national census. Eighty‐five percent of patients had hemophilia A, and 15% had hemophilia B. Regarding the degree of severity, those who had symptoms were those who went to the hospital; 65% were severe patients, and 22% were moderate patients. The mild population who does not present major symptoms are only identified once they attend the hospital for some type of surgery. As many as 53% of patients are over 18 years of age. However, in recent years, we have identified patients earlier; therefore, the number of patients under 18 years old is increasing and is now 47% of the population. Sixty‐two percent of the patients came from Lima and Callao, and only 38% were from other provinces. This is because people migrate to Lima and the cities on the coast precisely to receive a diagnosis, and therefore, they access treatment more than anywhere else. That is why we see the population concentrated especially in Lima.

### 4.9 Path forward

Working with the treatment centers in creating a network that will allow to start new lines of action to improve the care of PWH:

1. First, increasing the number of identified patients. This is key, since only 31% of them are presumably identified. A national census has been set up to this end. As a major issue for the state, the Ministry of Health is called to support this enterprise by providing professionals willing to move to the second phase of the project.
2. Second, we are improving the training of health professionals who work in first‐level health centers, especially those on the coast, which may be the first place where patients, especially children with symptoms, go for a diagnosis. By supporting these professionals in their educational program, the Peruvian Haematology Society is playing an important role to this end.
3. Third, establishing new treatment centers. Since there are regions in which no treatment center exists, it is critical to promptly identify PWH in those regions.
4. Fourth, establishing a national network to strengthen the quality of existing treatment centers. We are no longer talking as a system but as a country to balance access to the diagnosis and to the treatment. We have begun to work together, both with MINSA and EsSalud of hemophilia, as a common problem in Peru. Software that we are about to launch will be available in each treatment center to develop an actual real‐time database. The training of multidisciplinary teams cooperating with hematologists is also critical to improve the care of PWH. Finally, more reference laboratories are needed. Since we have serious problems with the laboratory diagnosis of deficiencies of individual clotting factors, VWF and inhibitors, new reference centers, one in the north and another in the south of the country, should be established, working in cooperation with the main reference center in Lima.
5. Fifth, access to treatment is key. There is an increase in clotting factor purchasing, according to the demand registered in recent years, which is also improving the doses for patients and the basic access to treatment. We need to standardize treatments, regardless of whether centers belong to the Ministry of Health or EsSalud. The national petitionary, which is critical, should be updated to allow the Ministry of Health to make purchases that cannot be made now, for example, concentrates of clotting factor IX, activated prothrombin complex, recombinant factor VII, VWD clotting factor, and emicizumab. We also want to improve access to desmopressin because it is not available for venous use, mainly due to logistical issues. Finally, we also need to consider other treatments based on a cost–benefit analysis.

### 4.10 Conclusions

We have improved during the last few years, but we are still facing big challenges. However, by unifying the professionals who work on this topic, we will further improve the care to our patients, not only for PWH but also, and even more, for those affected by VWD.

## 5 SITUATION IN ARGENTINA (DR. RAUL BORDONE)

### 5.1 Land

**FIGURE 5:**
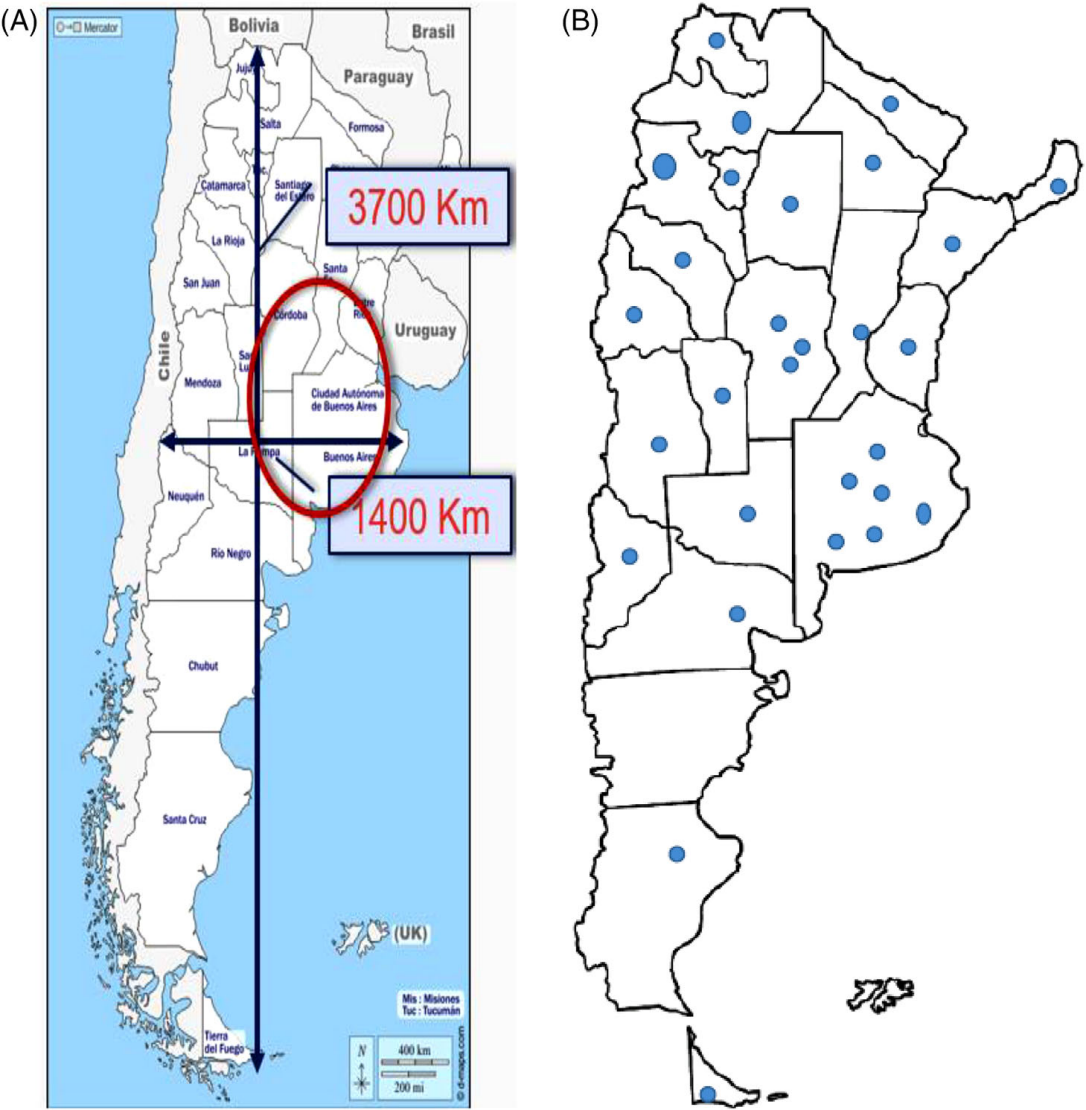
(A) Argentina: the land. (B) Hemophilia treatment centers as per the AAHDA (Asociación Argentina de Hemofilia y Desordenes Asociados)

Argentina is composed of 23 provinces in a federal district (Figure 4). It is an extremely vast country, measuring more than 3000 km in length and more than 1000 km in width (Figure 5). It has 45 million inhabitants, 48% men, and the population is mostly living in few areas. Almost 72% of the overall population lives in the province of Buenos Aires, and half of them live in the cities of Buenos Aires, Cordoba, and Santa Fe. (Figure 5)

### 5.2 Health care

Access to healthcare is quite complicated. Those who are covered by regular health insurance, which is called *union health insurance*, represent approximately 30% of national health coverage. Then, there are employees who depend on the provincial states that, depending on the province, cover 30%–50% of the population. Finally, there are those who depend directly on the nation, who are retired or pensioners, and armed forces, who constitute approximately 30% of the population. However, in reality, more than half of the population in Argentina does not have a permanent job, work, or are irregularly employed, and therefore, they do not have health insurance. Thus, having access to healthcare is quite difficult, and it falls onto the shoulders of the provincial states to cover those uninsured.

### 5.3 Hemophilia care

From the 2019 WFH survey, in Argentina, there are approximately 2800 registered PWH, of whom approximately 80% are treated on prophylaxis among those who are <21 years. This only represents 27% of the overall population of PWH, since the median age in the country is approximately 40 years old. Only 15% of the patients over 28 years of age are receiving prophylaxis, which implies that most of the patients who fulfil the criteria for receiving prophylaxis are not currently on treatment. In addition, according to this report and the documented prevalence, we believe the number of patients is underreported. It must be noted that the report made for the WFH is done by the Argentine Haemophilia Association, which does not represent all the centers currently active in Argentina. In Argentina, an adequate amount of clotting factors is forecasted, approximately 4.3 IU per capita, which makes Argentina one of the LATAM countries with the largest availability of clotting factors for the treatment of these patients. Most of them are plasma‐derived factors, and 40% are recombinant factors.

### 5.4 Hemophilia treatment centers

Currently, there are approximately 41 care centers in Argentina. Unfortunately, not all of them are treatment centers; most are hospital‐based, which leads to difficulties in many aspects of healthcare management. Fortunately, each province has at least a laboratory center for the assay of clotting factor VIII, a key availability to grant access to both the diagnosis and the possibility of carrying out personalized treatment.

### 5.5 Prophylaxis

Based on the lifestyle and the physical activity of the patient, we have been implementing personalized prophylaxis for several years based on population PK either by the WAPSS‐Heme or the myPKFiT platform. The widespread use of prophylaxis began in 2003, and most of our patients were on secondary or tertiary prophylaxis. We already have several patients in primary prophylaxis.

### 5.6 Challenges

We do not have official records, so we base cases on presumptions or partial records. Access to health care in general, especially for hemophiliac patients, is very inadequate. It depends on the centers, the resources they have and the provinces where they are located. Since most centers are hospital based, the time that health professionals devote to hemophilia cases is not a priority. Indeed, each clinician has to solve a variety of hematological pathologies that are often very pressing and demanding. Thus, in many centers, the time devoted to hemophilia is not adequate.

### 5.7 Path forward

In 2015, the process of joining several treatment centers to form a consolidated union was initiated. This process was achieved in 2020 by the creation of AAHDA, the *Argentine Association of Hemophilia Treatment Centres*, which allowed us to focus on several goals: create a registry to know exactly what is being done countrywide and to coordinate between centers, since there are many centers that do not have the resources and experience necessary, and others that care for only a few patients. The exchange of experiences and the training that have been implemented through this association of centers have helped to achieve several objectives, one of which was the creation of a biochemist network at the country level that could involve all biochemists from different centers. As a result, in each of the provinces, there is at least one laboratory where clotting factor assays can be carried out.

### 5.8 Von Willebrand disease

According to the WFH report, there are only approximately 400 cases diagnosed in Argentina, which is a substantially low number. This is because there are few laboratories that can assay VWF activity and antigen levels. Additionally, rather than by treatment centers, most patients with potentially confirmed diagnoses of VWD are managed by general hematologists. These patients are neither registered nor they have a treatment plan, especially before interventions, which causes a huge diversity of treatment from one center to another.

### 5.9 DDAVP

Currently, we only have access to the intravenous formulation. This makes the use of this drug difficult for us on an outpatient‐care basis, either for Von Willebrand or for mild hemophilia, where the outpatient use of DDAVP would be very beneficial. Although the use of DDAVP is included in the treatment guidelines for both VWD and hemophilia, it is mainly used as hormonal treatment in many *social projects* or *social health insurance*, and its administration cost is not fully covered. This implies that, at variance with clitting factor use, the patient must pay for a part of this medication. This further complicates its use. Additionally, because of the lack of ad hoc laboratory expertise, the very important use of the desmopressin response test is difficult to perform.

### 5.10 Conclusions

Argentina is fortunate to have many treatment centers with properly trained professionals spread throughout all of Argentina, in relatively close proximity, despite being a very large country. There is adequate availability of clotting factors for all patients, allowing for appropriate treatment. Availability to prophylaxis is variable according to the different circumstances, such as access to healthcare, but there is availability of prophylaxis to all patients. There are several centers that have routinely used personalized prophylaxis for several years, but unfortunately, a lack of records impairs the details on these patients.

## 6 SITUATION IN MEXICO (DR. CARLOS MARTINEZ MURILLO)

### 6.1 Hemophilia care

**FIGURE 6:**
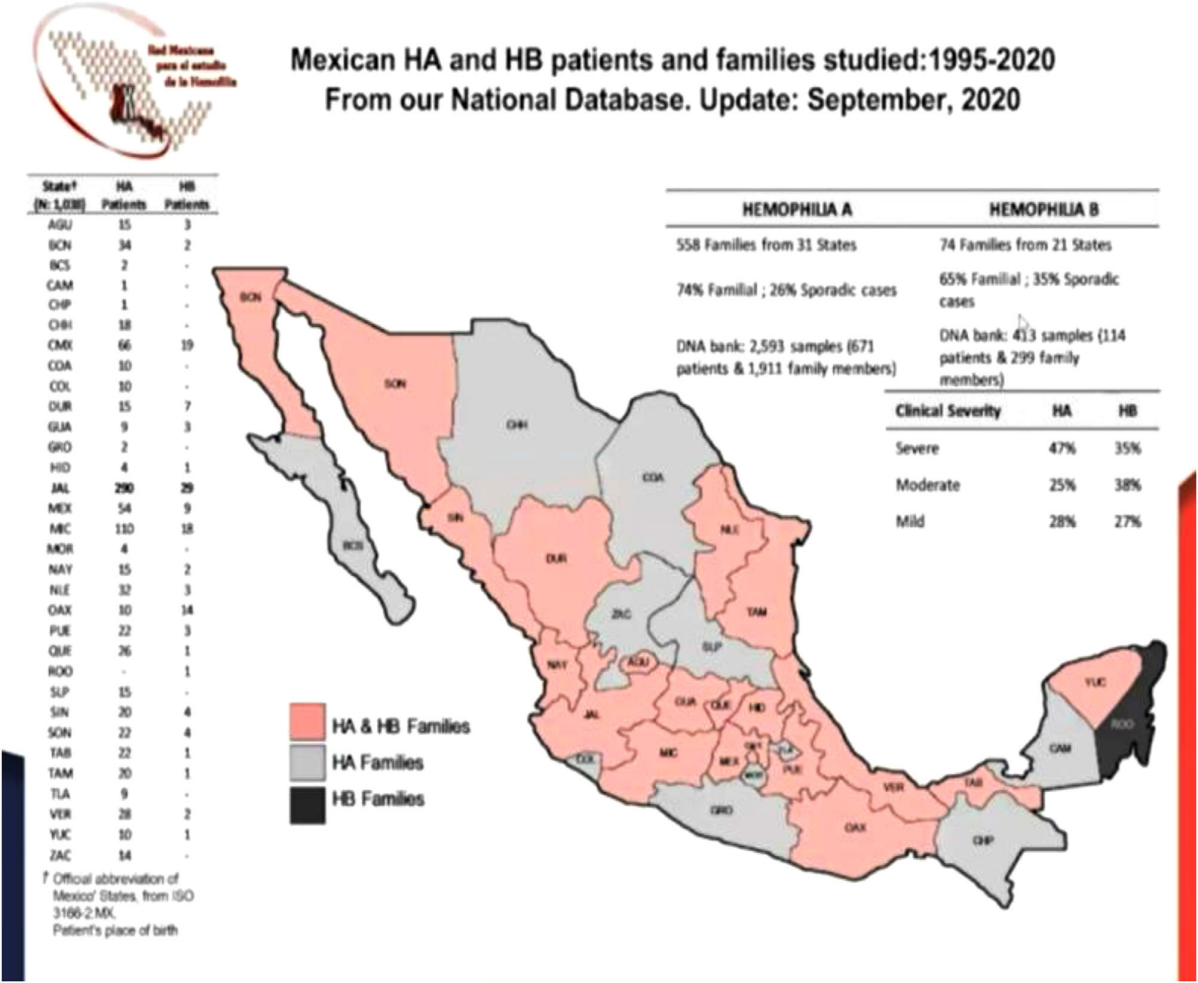
Mexico: the land and hemophilia family locations

Mexico is currently experiencing a transition regarding the treatment of hemophilia due to the improvement in the process of acquisition of clotting factor concentrates by the government. With an approximate population of 6192 patients with hemophilia (5853 with hemophilia A and 339 with hemophilia B) (Figure 6), the use of clotting factors in Mexico was approximately 1.8 IU per capita before 2019. Regarding the incidence of inhibitors, statistical data are variable and depend on the quality of the published epidemiological studies, which is why the figures vary between 10% and 26% with underreported cases. Most of these patients are treated with recombinant FVIIa concentrates and an activated prothrombin complex.

### 6.2 Treatment of severe hemophilia

Most of the pediatric population has been treated with prophylaxis using recombinant and plasma‐derived products for a few years. However, there are still several children who only receive treatment on demand. In 2019, a group of experienced doctors in treating hemophilia patients formed an advisory committee that collaborated with the secretary of the treasury of the federal government to establish new strategies to improve the quality of care in patients with hemophilia. After months of work carrying out a situational diagnosis and having meetings with the main pharmaceutical companies, the agreements and commitments of the three parties (federal government, medical group, and pharmaceutical industry) were consolidated.

### 6.3 Path forward

Established agreements: Mexico is in transition toward the consolidated purchase model and is working on a standardization of treatment throughout all institutions [7]. The per capita indicator should ideally be >4 IU, with an approximate acquisition of more than 550 million units (80% of the acquisitions of factor VIII products should be of recombinant origin, and the remaining ones should be plasma derived). In the case of factor IX, recombinant and plasma‐derived factors should be purchased. Plans to establish an interinstitutional medical registry regulated by health authorities should be implemented. Prophylaxis should be the standard of treatment, with priority to primary prophylaxis. The acquisition of emicizumab for 380 patients in 3 years, mainly for patients with hemophilia A and inhibitors. Better support in the diagnosis of suspected hemophilia, detection of inhibitors and PK as well as support to carry out a study whose aim is to identify the main genetic alterations (by random sampling) should be implemented. Promoting multicenter studies in Latin America through the creation of a Working Group in Mexico and Latin America (GLATHEM/OLAHH) would be ideal. Finally, one of the achievements of the medical community was the completion and recent publication of the Technical Protocol for Haemophilia Care [8], which standardizes the comprehensive management of patients with hemophilia regardless of the institution that treats them. This protocol includes all products to treat hemophilia A and B, as well as bypass agents and emicizumab. Based on this publication, access to factor concentrates is guaranteed for all patients, and the goal of >3 IU per capita is achieved. This will make it possible to standardize patient care for primary and secondary prophylaxis, as well as guarantee the use of desmopressin (DDAVP) and tranexamic acid.

### 6.4 Conclusions

In Mexico, efforts have been made toward a consolidated purchase system and additional services for the benefit of patients due to the efforts of a committee of advisers in hemophilia. This is a huge effort from the federal government and the clinicians who keep working to ensure that all the established agreements are met. The COVID‐19 pandemic has delayed the completion of established agreements. However, the work to fulfil them continues through the Special Program for Haemophilia Patient Care across the country, with the endorsement of health authorities. With such agreements and commitments, it is expected that Mexico will improve patient care in the medium term, with a significant improvement in international indicators as well.

## 7 OVERALL CONCLUSIONS

The vastness of the geographies of some of the countries and the available resources hampers progress in the care of persons with bleeding disorders in LATAM countries, as illustrated by this symposium. The information summarized emphasizes how the geography of a country and the different healthcare infrastructures play a major role in how care is offered. It also provides a path for other countries to evaluate these issues in their own realities. In parallel, these data provide hope to many developing countries; despite obstacles, strides can be made in the care of the bleeding disorder community.

## FUNDING INFORMATION

The authors received no specific funding for this work.

## CONFLICTS OF INTEREST

None of the authors received remuneration for the preparation of this manuscript. The views expressed in the manuscript are those of the authors alone. Giovanni G. DiMinno: Speaker or a member of a speaker bureau for Bayer, CSL Behring, Novo Nordisk, Pfizer, Sobi, Takeda; consultant or ad hoc speaker/consultant for Bayer, Novo Nordisk, Pfizer, Sanofi Aventis, Kedrion. Raul Bordone: Scientific advisory boards for CSL Behring, Novo Nordisk, Octapharma, Roche, Takeda; speaker honarariums from Kedrion, CSL Behring, Novo Nordisk, Octapharma, Roche, Takeda; support for Congress from Bayer, CSL Behring, Novo Nordisk, Octapharma, Pfizer, Roche, Takeda. Juan Carlos Beltran: Kedrion Colombia SAS. Prasad Mathew: Kedrion SpA.

## AUTHOR CONTRIBUTIONS

Giovanni G. DiMinno and Prasad Mathew wrote and finalized the paper. Lida Milena Araujo Cabrera, Nancy Loayza Urcia, Raul Bordone, and Carlos Martinez Murillo contributed to their individual country data and reviewed the final manuscript. Juan Carlos Beltran reviewed and supported the symposium through CLAHT.

## References

[1] Boadas A , Ozelo MC , Solano M , Berges A , Ruiz‐Saez A , Linares A , et al. Haemophilia care in Latin America; assessment and perspectives. Haemophilia. 2018;24(6):e395–401. 30144214 10.1111/hae.13607

[2] Foundation Hope and Life USA . A look at hemophilia in Latin American countries. https://fhlusa.org/a‐look‐at‐hemophilia‐in‐latin‐america. Accessed May 14, 2021.

[3] https://www.claht.org/web/index.php/eventos/eventos‐claht‐anteriores/congreso‐claht‐2021. Accessed October, 2021.

[4] Srivastava A , Santagostino E , Dougall A , Kitchen S , Sutherland M , Pipe SW et al. WFH guidelines for the management of hemophilia. 3rd ed. Haemophilia. 2020;26:1–158. 32744769 10.1111/hae.14046

[5] https://cuentadealtocosto.org/site/hemofilia/dia‐mundial‐de‐la‐hemofilia‐2021. Accessed January, 2022.

[6] Peyvandi F , Mannucci PM , Garagiola I , El‐Beshlawy A , Elalfy M , Ramanan V , et al. A randomized trial of factor VIII and neutralizing antibodies in hemophilia A. N Engl J Med. 2016;374:2054–64. 10.1056/NEJMoa1516437 27223147

[7] López‐Arroyo JL , Pérez‐Zúñiga JM , Merino‐Pasaye LE , Saavedra‐González A , Alcivar‐Cedeño LM , Álvarez‐Vera JL , et al. Consensus on Hemophilia in Mexico. Gac Med Mex. 2021;157:S1–S35. 10.24875/GMM.M21000463 33819260

[8] General Health Council . Technical protocol for the care of patients with haemophilia. 2022. http://www.csg.gob.mx/descargas/pdf/priorizacion/gastos‐catastroficos/protocolos/2021/Protocolo_Txcnico_D66x_D67xD68_HEMOFILIAV09122021FINALV322022.pdf. Accessed January, 2022.

